# Determining factors of thermoelectric properties of semiconductor nanowires

**DOI:** 10.1186/1556-276X-6-502

**Published:** 2011-08-19

**Authors:** Denis O Demchenko, Peter D Heinz, Byounghak Lee

**Affiliations:** 1 Department of Physics, Virginia Commonwealth University, Richmond, VA 23284, USA; 2 Department of Physics, Texas State University, San Marcos, TX 78666, USA

## Abstract

It is widely accepted that low dimensionality of semiconductor heterostructures and nanostructures can significantly improve their thermoelectric efficiency. However, what is less well understood is the precise role of electronic and lattice transport coefficients in the improvement. We differentiate and analyze the electronic and lattice contributions to the enhancement by using a nearly parameter-free theory of the thermoelectric properties of semiconductor nanowires. By combining molecular dynamics, density functional theory, and Boltzmann transport theory methods, we provide a complete picture for the competing factors of thermoelectric figure of merit. As an example, we study the thermoelectric properties of ZnO and Si nanowires. We find that the figure of merit can be increased as much as 30 times in 8-Å-diameter ZnO nanowires and 20 times in 12-Å-diameter Si nanowires, compared with the bulk. Decoupling of thermoelectric contributions reveals that the reduction of lattice thermal conductivity is the predominant factor in the improvement of thermoelectric properties in nanowires. While the lattice contribution to the efficiency enhancement consistently becomes larger with decreasing size of nanowires, the electronic contribution is relatively small in ZnO and disadvantageous in Si.

## Introduction

Using nanostructures for thermoelectric (TE) materials is a promising prospect as it opens up a possibility of controlling the TE properties by modifying the size and shape, in addition to the composition of the material. The TE properties of a material are characterized by a dimensionless figure of merit, ZT *= TS*^2^*σ*/(*κ_e _+ κ_l_*), where *T, S, σ, κ_e_*, and *κ_l _*are temperature, Seebeck coefficient (thermopower), electrical conductivity, electronic thermal conductivity, and lattice thermal conductivity, respectively. The main issue in finding large ZT materials is to balance the advantageous material properties, i.e., *S *and *σ*, with the detrimental *κ_l _*and *κ_e_*. The *S, σ*, and *κ_e _*are related to the electronic states and cannot be controlled independently in bulk materials. The *κ_l _*of bulk materials is a structural property that is also difficult to manipulate. It was suggested that reduction of the dimensionality should provide certain controllability over the individual transport coefficients, leading to a dramatic increase in ZT [1]. Bi_2_Te_3_/Sb_2_Te_3 _epitaxial superlattices were reported to exhibit a very high ZT of 2.4 at room temperature [2], larger than the maximum ZT of 1.14 for a *p*-type (Bi_2_Te_3_)_0.25_(Sb_2_Te_3_)_0.72_(Sb_2_Se_3_)_0.03 _alloy at room temperature [3], affirming the basic premise. In many cases, however, the decrease in phonon transport also leads to the reduction in power factor due to decrease in electron transport. The search for the balance between these factors is currently ongoing.

In this letter, we report the results of a theoretical investigation of TE properties of semiconductor nanowires to provide a microscopic picture for key contributing factors to improve the TE performance of nanostructures. The focus of this work is to distinguish electronic and lattice contributions to the enhancement of ZT in nanowires (NWs) by means of parameter-free calculations. Previous theoretical studies have provided proof-of-principle verification of ZT improvement in nanostructures [4,5], but their analyses were based on the lattice thermal conductivity speculated from the bulk value. By combining molecular dynamics (MD), density functional theory (DFT), and Boltzmann transport equation (BTE) methods, we compute all of the transport coefficients without empirical parameters.

## Methods of calculations

In this work, we used equilibrium MD simulations based on Green-Kubo formula, using LAMMPS [6,7] code to compute lattice part of thermal conductivity. The interatomic interactions in ZnO are modeled using the Buckingham potential with long-range Coulomb interactions [8,9]. This potential has been applied to describe elastic properties of ZnO nanostructures [10], their structural transformations [11,12], and thermal properties [13-15]. In case of Si NW, we used Stillinger-Weber [16] potential, which has been applied to model thermoelectric properties of Si both in the bulk and on the nanoscale [17,18]. The atomic and electronic structures of ZnO NW were calculated using planewave DFT method implemented in VASP code [19]. We used the generalized gradient approximation with Perdew-Burke-Ernzerhof exchange-correlation functional [20]. The *k*-point grid of 4 × 4 × 24 and an energy cutoff of 400 eV were used for all calculations. The TE coefficients of ZnO nanowires were calculated based on Boltzmann transport theory, using BoltzTraP [21]. The method calculates TE properties using the result of *ab initio *electronic structure method results. Within this semi-classical method, the group velocity and the mass tensor are calculated using the DFT band dispersion. Assuming a constant carrier relaxation time, one can calculate the TE coefficients from the transport tensors [22].

The only physical parameter that we do not seek to calculate from first principles is the relaxation time, *τ*. It is a complex function of atomic and electronic structure, temperature, impurities, and carrier concentrations. Experimentally, it has been reported that the carrier relaxation time in ZnO nanowires is close to the bulk relaxation time. The time-resolved terahertz spectroscopy measurements of electron conductivity yielded the relaxation time of 28 and 35 fs for 500-nm radius nanowires and films, respectively, at the electron density of 10^17 ^cm^-3 ^[23]. Throughout this paper, we adopt the ZnO bulk relaxation time *τ *= 30 fs for our calculations. The relaxation time in NW is generally larger than in the bulk [4], and therefore, our estimation of *τ *represents a correct quantitative picture. In the case of Si NW, we also used the carrier concentration-dependent *τ *fitted into the experimental data [24].

## Results and discussion

**Figure 1:**
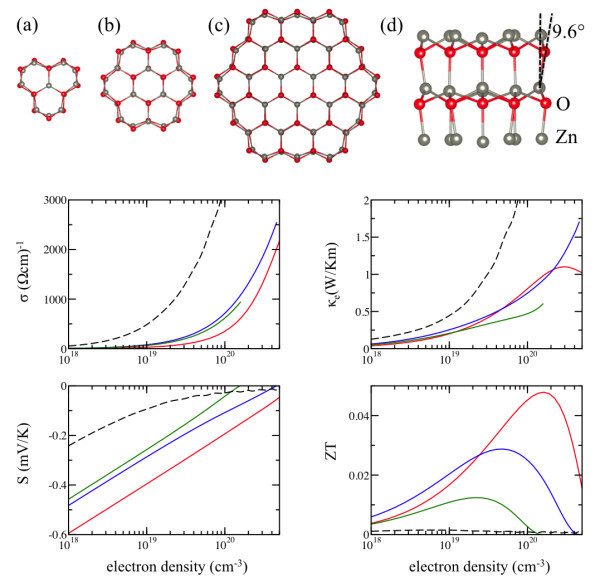
**Relaxed atomic structure and room-temperature transport coefficients and ZT of ZnO NWs**. (a), (b), and (c) shows the [0001] cross section of 8-, 10-, and 17-Å-diameter NWs, respectively. Grey spheres denote Zn atoms and red spheres indicate O atoms. (d) shows the side view of 8-Å-diameter NW. In the transport coefficients plots, the red, blue, and green lines denote the results for NW of diameter 8, 10, and 17 Å, respectively. The bulk values (black dashed lines) are shown for comparison. (Color online).

When the ZnO NW is grown in [0001] direction, the surface oxygen atoms move outward away from the nanowire center while the zinc atoms moving inward toward the center (Figure 1 (a-d)). This "buckling" of the surface atoms has been reported in elastic low-energy diffraction experiments, where Zn-O bonding on the surface of ZnO (10-10) is tilted by an angle of 12° from the [0001] axis [25]. In our calculations, the tilting angle is 9.6°, in agreement with previous DFT results [26]. We also find that the bond length of the O-Zn surface dimer is reduced from 2.07 to 1.9 Å (similar to Ref. [27]). This surface reconstruction removes the surface states from the nanowire band gap. The computed electronic band gaps are 1.65, 1.47, and 1.13 eV for 8, 10, and 17 Å NWs, showing gap opening due to the quantum confinement. In comparison, the bulk ZnO band gap in our calculations is 0.73 eV.

The electronic structure and thermoelectric properties of Si [001] NW were computed for comparison. The diameter of the NW was 12 Å with single-bonded surface atoms removed and the surface atoms passivated by hydrogen to remove dangling bonds (inset to Figure 3). The computed band gap in the Si NW was 2.11 eV, in comparison with computed 0.56 eV in the bulk. The electronic properties of Si NWs have been studied in great detail in the past [28]. Electronic part of thermoelectric properties has also been addressed previously [4], suggesting improvement of ZT to as large as 1.55. However, the lattice thermal conductivity in these calculations was presumed instead of being calculated. The significance of the lattice conductivity is discussed below.

The heat transport through the phonon channels in nanowires is analyzed here via the classical MD simulations. The atomic structures for ZnO NWs used in MD calculations are the same as in the electronic part calculations, with their dimension in [0001] direction extended to 50 Å. Using periodic boundary conditions in this directions, our tests show that for these NWs, the effects of finite length are negligible. The value of the lattice thermal conductivity is the averaged integral of the heat current autocorrelation function (ACF). In bulk ZnO and ZnO NWs, the heat current ACF exhibits strong oscillations due to zero wave vector optical phonon modes. For example, ACF for the 8 Å ZnO NW shows a 56.7-meV phonon mode, which agrees well with the energy (approximately 53 meV) of the optical branch at the Γ-point for bulk wurtzite ZnO [29]. Although these optical phonon oscillations do not contribute to the heat current, they significantly complicate the numerical integration of the heat current ACF [30]. To avoid numerical instabilities due to this optical phonon mode, a very small MD step of 0.1 fs was taken in our MD calculations. *κ_l _*were averaged over the total MD run times of 3 ns. At room temperature, the experimental *κ_l _*of bulk ZnO varies from about 50 to 140 W/Km [31,32], depending on growth conditions. The bulk values of *κ_l _*= 85 W/Km in [0001] direction were obtained by extrapolating to infinity the results for a series of simulation box sizes. For the NWs, a significant reduction in *κ_l _*is obtained. The converged values of the *κ_l _*at room temperature are 7.9, 9.2, and 11.7 W/Km for 8, 10, and 17 Å NWs, respectively. In case of Si NW, there are no optical phonon oscillations in the ACF and computing *κ_l _*is relatively straightforward. In the bulk Si, we obtain *κ_l _*= 254 W/Km at room temperature. This value is overestimated by about 70% in comparison with experiment, which is typical for Stillinger-Weber potential. In the 12 Å Si NWs, *κ_l _*is reduced to 2.8 W/Km. This value is close to the presumed lattice thermal conductivity in the previous study by Vo et al. [4]. This reduction of *κ_l _*can stem from several processes, such as three-phonon Umklapp, mass difference, and boundary/surface scattering. In ZnO and Si NWs considered here, the primary process is likely the increased surface phonon scattering, evident from the fact that *κ_l _*decreases with increasing surface to volume ratio.

The calculated TE properties of ZnO NWs at room temperature are shown in Figure 1 as a function of carrier concentration. Compared with the bulk TE coefficients, *σ *changes are unfavorable to the ZT while *S *and *κ_e _*change positively. Notice that the unfavorable change in *σ *is much larger than the favorable increase in the magnitude of *S*, but the quadratic dependence of ZT on *S *nearly cancels the effect of linear dependence on *σ*. The combination of these changes, together with the change in *κ_l_*, conspires a significantly improved ZT in NWs. The changes of individual TE transport coefficients are consistent with the tendencies found in previous calculations of Si NW [4], nanoporous Si [5], and Si NW computed here (not shown). The maximum ZT is more than 30 and 20 times larger than that of the bulk in ZnO and Si, respectively, confirming the basic proposition of ZT enhancement in nanostructures. However, both Si and ZnO nanowires considered here are ultra thin; therefore, these results represent the largest values for ZT enhancements possible solely due to scaling down the sizes and using these materials on the nanoscale.

**Figure 2:**
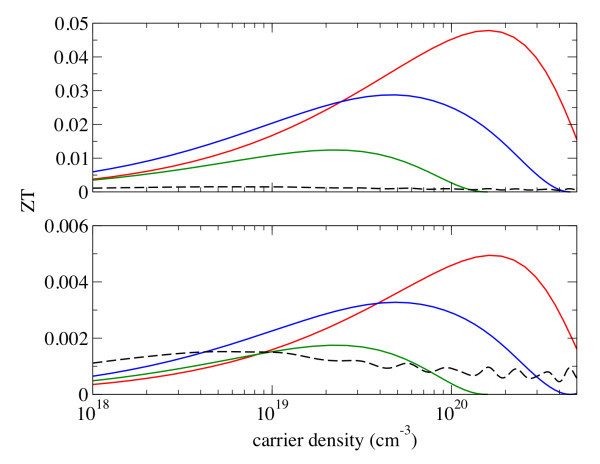
**ZT of ZnO NWs of 8 &#197 (red), 10 &#197 (blue), and 17 &#197 (green) in diameter.** The bulk values (black dashed lines) are shown for comparison. Top panel: The lattice contribution of thermal conductivity obtained from the molecular dynamics calculations is used. Bottom panel: The lattice contribution of thermal conductivity of bulk ZnO (85 W/Km) is used for the calculation of the NW ZT. (Color online).

To distinguish the electronic and lattice contribution to the improvement of ZT separately, we computed the ZT of NWs using the lattice thermal conductivity of bulk ZnO, *κ_l_*^bulk^, and that of NWs, *κ_l_*^MD^. The top panel of Figure 2 shows the ZT of ZnO NW using *κ_l_*^MD ^of each NW and the bottom panel shows the *ZT *using *κ_l_*^bulk^. While a large enhancement of maximum ZT in NW has contributions from both electronic and lattice part (top panel), the increase in ZT at the bottom panel stems solely from the changes in the electronic structure. In Table 1 we show the ratio of the maximum figure-of-merit of nanowire, ZT*, to the bulk counterpart, ZT*_bulk_: *ζ *= ZT*/ZT*_bulk_. The enhancement of ZT due to the changes in electronic structure can be parameterized by *ζ_e _*= ZT*(*κ_l_*^bulk^)/ZT*_bulk_, where ZT*(*κ_l_*^bulk^) is the ZT of NWs calculated using the bulk lattice thermal conductivity. It appears that the quantum confinement has only modest effects, i.e., *ζ_e _*= 3.25, 2.15, and 1.15 for 8, 10, and 17 Å nanowires, respectively. The remaining contribution of *ζ *comes from the lattice part, *ζ_l _= ζ/ζ_e_*. We see a much larger effect of lattice contribution, i.e., *ζ_l _*= 9.69, 8.79, and 7.10 for the same set of NWs. We also observe that both contributions of the electronic and the lattice part to the enhancement of ZT decrease as the diameter of the NW increases. In particular, the electronic contribution to the ZT enhancement becomes negligible when the NW diameter is as large as 17 Å.

**Table 1:** Enhancement of ZT in ZnO and Si NW

	NW diameter (Å)			
	ZnO			Si
	8	10	17	12
*ζ = *ZT*/ZT*_bulk_	31.5	18.9	8.17	20.5
*ζ_e _= *ZT*(*κ_l_*^bulk^)/ZT*_bulk_	3.25	2.15	1.15	0.264
*ζ_l _*= *ζ/ζ_e_*	9.69	8.79	7.10	77.7

*ζ *= ZT*/ZT*_bulk _is the ratio of the maximum ZT of NW to maximum ZT of bulk ZnO. *ζ_e _*and *ζ_l _*are electronic and lattice contribution to *ζ*.

**Figure 3:**
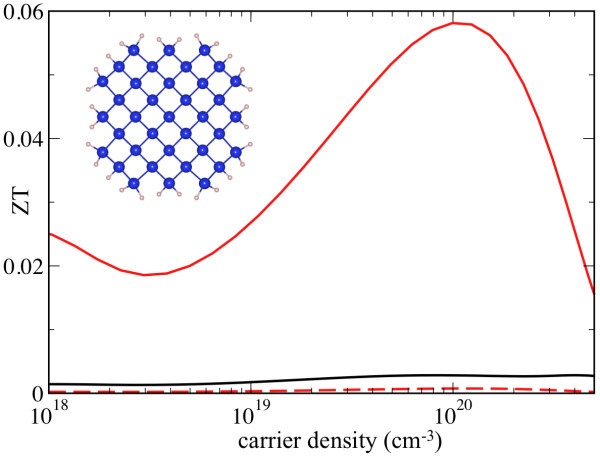
**ZT of 12-&#197-diameter Si NW's (red) and bulk Si (black).** The dashed (red) line is the ZT of the same Si NW calculated with bulk lattice thermal conductivity. The inset shows the cross-section atomic configuration of the NW. The NW axis direction is [001]. The lattice contributions of thermal conductivity of bulk Si and the NW, 254 and 2.8 W/Km, respectively, were derived from MD calculations. (Color online).

This enhancement of ZT due to the reduction of the lattice thermal conductivity is even more pronounced in Si NWs (Figure 3). As presented in Table 1 the changes in the electronic structure on the nanoscale lead to the reduction of ZT by nearly a factor of 4, with *ζ_e _*= 0.264, in contrast to those of ZnO. This reduction stems from the decreased electric conductivity in comparison with the bulk. The thermopower, on the other hand, remains relatively unchanged. As a result, the power factor in Si nanowires is about 4.6 times smaller than that in the bulk. Nevertheless, the reduction in the lattice thermal conductivity is by almost a factor of 90 leads to the more than 70-fold lattice contribution to the enhancement of ZT. (Note that the lattice thermal conductivity of Si nanowires is highly anisotropic and very sensitive to the geometry of the surface.)

Finally, we note that the maximum values of ZT we obtain for ZnO NW is approximately 0.05 and similarly it is approximately 0.06 in Si NW. This is much smaller than the previously calculated value in Si NW, approximately 1.55 [4]. The differences between these calculations appear to arise from the differences in computed electric conductivity and consequently power factors. The values of electric conductivity for bulk Si computed here are in agreement with that of the experiment [23] for electron concentrations between 10^17 ^to 10^20 ^cm^-3 ^as well as previous calculations (Ref. [5]), while it appears to be overestimated in Ref. [4]. The values of ZT computed here suggest that reducing the scale of nanostructures alone does not improve the ZT to a practical degree. Our findings indicate that other mechanisms must be involved in the improvement of ZT, in addition to using nanocrystals. For example, in recent experiments, the enhanced ZT values in nanowires were obtained due to surface roughness and impurity scattering [33,34].

## Conclusion

In conclusion, we presented the results of BTE calculations of ZnO and Si NWs based on electronic and lattice properties computed using DFT and classical MD, respectively. Not all electronic and lattice thermal coefficients change favorably when size of the nanowire is reduced. By decoupling the effects of electronic and lattice thermal conductivity on ZT values of semiconductor nanowires, we found that the reduction in *κ_l _*plays a predominant role in the enhancement of ZT. For example, for the 8 Å ZnO and 12 Å Si nanowire, this enhancement is by a factor of 10 and 78, respectively. We conclude that for ZnO nanowires with diameter of 17 Å and larger, the lattice thermal conductivity contribution to the enhanced ZT is overwhelmingly more important than that of the electrons. Opposite to the common belief, the electronic contribution changes disadvantageously in Si NWs. In both materials, although the improvement in ZT is substantial in comparison with the bulk, the ZT of about approximately 0.05 are the maximal achievable values as a result of scaling down of the materials to the nanoscale level (down to about 10 Å).

## Competing interests

The authors declare that they have no competing interests.

## Authors' contributions

DOD carried out molecular dynamics calculations and some of the density functional theory (DFT) calculations. PDH and BL performed DFT calculations of the electronic part of thermal conductivity. All authors read and approved the final manuscript.

## References

[B1] Hicks L Dresselhaus M Effect of quantum-well structures on the thermoelectric figure of merit Phys Rev B 1993 47 12727 12731 10.1103/PhysRevB.47.12727 10005469

[B2] Venkatasubramanian R Siivola E Colpitts V O'Quinn B Thin-film thermoelectric devices with high room-temperature figures of merit Nature 2001 413 597 602 10.1038/35098012 11595940

[B3] Ettenberg MH Jesser WA Rosi FD Caillat T A new n-type and improved p-type pseudo-ternary (Bi_2_Te_3_)(Sb_2_Te_3_)(Sb_2_Se_3_) alloy for Peltier cooling Proceedings of 15th International Conference on Thermoelectrics 1996 Piscataway: IEEE 52 56

[B4] Vo T Williamson AJ Lordi V Galli G Atomistic design of thermoelectric properties of silicon nanowires Nano Letters 2008 8 1111 1114 10.1021/nl073231d 18302325

[B5] Lee J-H Galli GA Grossman JC Nanoporous Si as an efficient thermoelectric material Nano Letters 2008 8 3750 3754 10.1021/nl802045f 18947211

[B6] Plimpton SJ Fast parallel algorithms for short-range molecular dynamics J Comp Phys 1995 117 1 19 10.1006/jcph.1995.1039

[B7] LAMMPS Molecular Dynamics Simulator http://lammps.sandia.gov

[B8] Binks DJ Grimes RW Incorporation of monovalent Ions in ZnO and their influence on varistor degradation J Am Ceram Soc 1993 76 2370 2372 10.1111/j.1151-2916.1993.tb07779.x

[B9] Grimes RW Binks DJ Lidiard AB The exent of zinc-oxide solution in zinc chromate spinel Philos Mag A 1995 72 651 668 10.1080/01418619508243791

[B10] Agrawal R Peng B Gdoutos EE Espinosa HD Elasticity size effects in ZnO nanowires - a combined experimental-computational approach Nano Letters 2008 8 3668 3674 10.1021/nl801724b 18839998

[B11] Dai L Cheong WCD Sow CH Lim CT Tan VBC Molecular dynamics simulation of ZnO nanowires: size effects, defects, and super ductility Langmuir 2010 26 1165 1171 10.1021/la9022739 19711920

[B12] Kulkarni AJ Zhou M Sarasamak K Limpijumnong S Novel phase transformation in ZnO nanowires under tensile loading Phys Rev Lett 2006 97 105502 105506 17025826 10.1103/PhysRevLett.97.105502

[B13] Kulkarni AJ Zhou M Tunable thermal response of ZnO nanowires Nanotechnology 2008 18 435706 435712

[B14] Kulkarni AJ Zhou M Surface-effects-dominated thermal and mechanical responses of zinc oxide nanobelts Acta Mech Mech Sinica 2006 22 217 224 10.1007/s10409-006-0111-9

[B15] Kulkarni AJ Zhou M Size-dependent thermal conductivity of zinc oxide nanobelts Appl Phys Lett 2006 88 141921 141924 10.1063/1.2193794

[B16] Stillinger FH Weber TA Computer simulation of local order in condensed phases of silicon Phys Rev B 1985 31 5262 5271 10.1103/PhysRevB.31.5262 9936488

[B17] Volz SG Chen G Molecular dynamics simulation of thermal conductivity of silicon nanowires Appl Phys Lett 1999 75 2056 2058 10.1063/1.124914

[B18] Volz SG Chen G Molecular-dynamics simulation of thermal conductivity of silicon crystals Phys Rev B 2000 61 2651 2656 10.1103/PhysRevB.61.2651

[B19] Kresse G Furthmüller J Efficient iterative schemes for *ab initio *total-energy calculations using a plane-wave basis set Phys Rev B 1996 54 11169 11186 10.1103/PhysRevB.54.11169 9984901

[B20] Perdew JP Burke K Ernzerhof M Generalized gradient approximation made simple Phys Rev Lett 1996 77 3865 3868 10.1103/PhysRevLett.77.3865 10062328

[B21] Madsen GKH Singh DJ BoltzTraP: a code for calculating band-structure dependent quantities Comp Phys Comm 2006 175 67 71 10.1016/j.cpc.2006.03.007

[B22] Ashcroft NW Mermin ND Solid State Physics New York: Holt, Rinehart, and Winston 1976

[B23] Baxter J Schmuttenmaer CA Conductivity of ZnO nanowires, nanoparticles, and thin films using time-resolved terahertz spectroscopy J Phys Chem B 2006 110 25233 25239 10.1021/jp064399a 17165967

[B24] Gaymann A Geserich HP Van Löhneysen H Temperature dependence of the far-infrared reflectance spectra of Si: P near the metal-insulator transition Phys Rev B 1995 52 16486 16493 10.1103/PhysRevB.52.16486 9981047

[B25] Duke CB Meyer RJ Paton A Mark P Calculation of low-energy-electron-diffraction intensities from ZnO(1010). II. Influence of calculational procedure, model potential, and second-layer structural distortions Phys Rev B 1978 18 4225 4240 10.1103/PhysRevB.18.4225

[B26] Shen X Pederson MR Zheng J-C Davenport JW Muckerman JT Allen PB http://arxiv.org/pdf/cond-mat/0610002v1 10.1021/nl070788k 17608442

[B27] Wang B Zhao J Jia J Shi D Wan J Wang G Structural, mechanical, and electronic properties of ultrathin ZnO nanowires Appl Phys Lett 2008 93 021918 021921 10.1063/1.2951617

[B28] Vo T Williamson AJ Galli G First principles simulations of the structural and electronic properties of silicon nanowires Phys Rev B 2006 74 045116 045128

[B29] Serrano J Manjón FJ Romero AH Ivanov A Lauck R Cardona M Krisch M The phonon dispersion of wurtzite-ZnO revisited Physica Status Solidi (B) 2007 244 1478 1482 10.1002/pssb.200675145

[B30] Landry ES Hussein MI McGaughey AJH Complex superlattice unit cell designs for reduced thermal conductivity Phys Rev B 2008 77 184302 184315

[B31] Wolf MW Martin JJ Low temperature thermal conductivity of zinc oxide Physica Status Solidi (A) 1973 17 215 220 10.1002/pssa.2210170124

[B32] Özgür Ü Gu X Chevtchenko S Spradlin J Cho SJ Morkoc H Pollak FH Everitt HO Nemeth B Nause JE Thermal conductivity of bulk ZnO after different thermal treatments J Electronic Materials 2006 35 550 555 10.1007/s11664-006-0098-9

[B33] Boukai AI Bunimovich Y Tahir-Kheli J Yu J-K Goddard WA III Heath JR Silicon nanowires as efficient thermoelectric materials Nature 2008 451 168 171 10.1038/nature06458 18185583

[B34] Hochbaum AI Chen R Delgado RD Liang W Garnett EC Najarian M Majumdar A Yang P Enhanced thermoelectric performance of rough silicon nanowires Nature 2008 451 163 167 10.1038/nature06381 18185582

